# Assessment of Community-Level Disparities in Coronavirus Disease 2019 (COVID-19) Infections and Deaths in Large US Metropolitan Areas

**DOI:** 10.1001/jamanetworkopen.2020.16938

**Published:** 2020-07-28

**Authors:** Samrachana Adhikari, Nicholas P. Pantaleo, Justin M. Feldman, Olugbenga Ogedegbe, Lorna Thorpe, Andrea B. Troxel

**Affiliations:** 1 Department of Population Health, New York University Grossman School of Medicine; 2 Department of Medicine, New York University Grossman School of Medicine

## Abstract

This cross-sectional study examines the association of neighborhood race/ethnicity and poverty with coronavirus disease 2019 (COVID-19) infections and related deaths in urban US counties.

## Introduction

Urban counties in large metropolitan areas in the United States are among the most affected by the coronavirus disease 2019 (COVID-19) pandemic, with high proportions of confirmed infection among those who have been tested.^1^ While there is growing evidence of disparities by race/ethnicity across neighborhoods,^2,3^ the extent to which neighborhood poverty is associated with infection and deaths is not clear. In this cross-sectional study, we examined the association of neighborhood race/ethnicity and poverty with COVID-19 infections and related deaths in urban US counties, hypothesizing disproportionate burdens in counties with a larger percentage of the population belonging to minority racial/ethnic groups and a higher rate of poverty. This study is among the first to investigate such associations in US metropolitan areas.

## Methods

### Data Sources and Variables

Counties were grouped by US Office of Management and Budget–defined combined statistical areas (CSAs).^4^ We included CSAs centered on the following 10 major US cities, all of which experienced early surges of COVID-19 infections: New York, New York; Boston, Massachusetts; New Orleans, Louisiana; Detroit, Michigan; Los Angeles, California; Atlanta, Georgia; Miami, Florida; Chicago, Illinois; Philadelphia, Pennsylvania; and Seattle, Washington. Combined, these CSAs accounted for 63.5% of confirmed COVID-19 infections (834 126 of 1 312 679) in the United States as of May 10, 2020. We included 158 counties that experienced at least 1 death attributed to confirmed COVID-19.

Information regarding county-level poverty rates and median household income was obtained from the 2018 US Census Small Area Income and Poverty Estimates Program.^5^ We calculated county proportions of racial/ethnic minority groups (per US Census methodology, individuals who did not identify as non-Hispanic White alone) using the 2018 US Census Annual Estimates of the Resident Population.^5^ We also obtained cumulative COVID-19 infection and death data, made publicly available by the Centers for Disease Control and Prevention as well as state health departments and aggregated by USA Facts^6^ on May 11, 2020. Per the Common Rule, institutional review board approval was unnecessary because of the use of publicly available, deidentified data. This study followed the Strengthening the Reporting of Observational Studies in Epidemiology (STROBE) reporting guideline for cross-sectional studies.

### Statistical Analysis

Cumulative COVID-19 incident infections and deaths were linked with population-level and community-level variables by unique county federal information processing standards codes. We calculated cumulative incidence, ie, the total number of COVID-19 infections and deaths per 100 000 individuals (hereafter, infection and death rates) using the population of each county as the denominator. Quartiles for percentage of residents from racial and ethnic minority groups were calculated for the 158 counties, ranging from substantially White (3.0%-17.9%) to less diverse (18.0%-29.4%), more diverse (29.5%-44.5%), and substantially non-White (>44.5%). The percentage of individuals living below the poverty level was binarized at the median poverty rate of the data set (ie, 10.7%) into less-poverty counties and more-poverty counties.

We summarized county-level characteristics for each CSA. To model the association between community factors and COVID-19 infection and death rates, we fitted random-effect linear regression models on the logarithm of infection and death rates with CSA-level random intercepts to account for clustering of counties within CSAs and including coefficients for poverty and race/ethnicity categories as well as their interactions. We compared model-adjusted rates for each race/ethnicity category among less-poverty counties and more-poverty counties. Statistical software R version 3.6.3 (R Project for Statistical Computing) package lme4 was used for the analyses. All statistical tests were 2-tailed, with a significance threshold of *P* < .05.

## Results

Table 1 summarizes the counties within the CSAs. Of 158 counties, 81 (51.3%) were considered less-poverty counties and 77 (48.7%), more-poverty counties. For the less-poverty counties, the median county-level income was $79 834 (range, $53 060-$119 731) compared with $60 240 (range, $36 850-$88 960) for the more-poverty counties. Infection rates for COVID-19 ranged from 41.7 to 3821.8 per 100 000 individuals, and death rates ranged from 0.34 to 271.3 per 100 000 individuals.

**Table 1.** Summary of County-Level Poverty, Income, Race/Ethnicity, and COVID-19 Infections and Deaths per 100 000 Residents, by CSA

CSA (No. of counties)	Median (range)				
	Population below poverty, %	County-level income, $	Population belonging to minority racial/ethnic group, %	Cumulative COVID-19 incident infections per 100 000 residents	Cumulative COVID-19 deaths per 100 000 residents
Atlanta (28)	12.4 (5.0-24.4)	62 554 (38 886-105 921)	34.1 (6.3-89.6)	259.4 (101.3-984.1)	7.6 (2.7-68.2)
Boston (19)	8.0 (5.3-17.5)	76 373 (56 141-100 374)	16.3 (7.7-55.3)	491.3 (147.0 -1900.6)	31.8 (1.2-92.0)
Chicago (17)	9.4 (4.2-16.2)	66 720 (53 060-93 540)	24.4 (3.0-58.3)	457.7 (52.9-1022.4)	16.7 (1.9-62.4)
Detroit (10)	10.7 (5.0-21.7)	62 356 (46 440-84 048)	17.4 (5.8-50.7)	290.0 (129.0-1033.2)	27.6 (2.0-119.9)
Los Angeles (5)	12.7 (9.1-14.9)	67 986 (63 310-89 373)	64.8 (55.4-74.4)	136.0 (78.7-315.5)	5.2 (2.2-15.2)
Miami (6)	12.1 (10.7-16.0)	57 714 (52 043-61 735)	44.0 (22.0-88.4)	208.5 (62.5-515.5)	10.4 (3.7-17.9)
New Orleans (11)	17.9 (11.8- 24.6)	50 830 (36 850-67 585)	37.3 (18.3-69.4)	1085.9 (340.3 -1869.9)	73.3 (20.8-177.4)
New York City (36)	10.6 (4.4-27.3)	79 662 (38 566-119 731)	36.7 (5.9-91.8)	1317.8 (43.6-3821.8)	93.5 (1.4-271.3)
Philadelphia (18)	10.8 (5.7-24.3)	68 348 (46 149-99 224)	27.6 (14.7-65.7)	606.5 (171.3-961.6)	34.9 (12.6 -69.7)
Seattle (8)	8.9 (7.5-13.7)	73 711 (58 239-94 822)	25.4 (19.6-40.8)	188.5 (41.6-326.1)	8.4 (0.3-22.2)

Abbreviation: COVID-19, coronavirus disease 2019; CSA, combined statistical area.

Model-adjusted rates and rate ratios (RRs) are reported in Table 2. In more-poverty counties, those with substantially non-White populations had an infection rate nearly 8 times that of counties with substantially White populations (RR, 7.8; 95% CI, 5.1-12.0) and a death rate more than 9 times greater (RR, 9.3; 95% CI, 4.7-18.4). Indeed, we found that among both more-poverty and less-poverty counties, those with substantially non-White or more diverse populations had higher expected cumulative COVID-19 incident infections compared with counties with substantially White or less-diverse populations (eg, more diverse counties with less poverty: RR, 3.2; 95% CI, 2.3-4.6). Similar associations were observed for deaths (eg, more diverse counties with less poverty: RR, 3.8; 95% CI, 2.2-6.7).

**Table 2.** Model-Adjusted Expected Rates and Rate Ratios for Coronavirus Disease 2019 Infection and Death Rates

County	Cumulative incident infections per 100 000 residents				Cumulative deaths per 100 000 residents			
	Model-adjusted rate		Rate ratio (95% CI)		Model-adjusted rate		Rate ratio (95% CI)	
	Less-poverty county	More-poverty county	Less-poverty county	More-poverty county	Less-poverty county	More-poverty county	Less-poverty county	More-poverty county
Substantially White	169.3	84.7	1 [Reference]	1 [Reference]	8.2	3.3	1 [Reference]	1 [Reference]
Less diverse	321.7	321.7	1.9 (1.4-2.6)	3.8 (2.3-6.1)	14.8	15.4	1.8 (1.1-3.0)	4.7 (2.2-10.0)
More diverse	541.8	433.4	3.2 (2.3-4.6)	5.1 (3.3-8.0)	31.2	16.2	3.8 (2.2-6.7)	4.9 (2.4-9.7)
Substantially non-White	474.0	663.7	2.8 (1.8-4.4)	7.8 (5.1-12.0)	21.3	30.7	2.6 (1.1-6.5)	9.3 (4.7-18.4)

## Discussion

While the excess burden of both infections and deaths was experienced by poorer and more diverse areas, racial and ethnic disparities in COVID-19 infections and deaths existed beyond those explained by differences in income. Lack of access to disaggregated data precludes us from further exploring causal mechanisms, such as structural racism or other social drivers. Studies that leverage community information with individual-level health data are likely to provide additional insights.
